# Enterococcus faecalis Endocarditis of All Four Native Valves: A Case Report

**DOI:** 10.7759/cureus.53837

**Published:** 2024-02-08

**Authors:** Alana Pinheiro Alves, Juliana Overbey, Max Jin, Eric Edewaard

**Affiliations:** 1 Internal Medicine, Western Michigan University Homer Stryker M.D. School of Medicine, Kalamazoo, USA

**Keywords:** case report, enterococcus bacteremia, transesophageal echocardiogram (tee), multi-valve endocarditis, infective endocarditis

## Abstract

*Enterococcus faecalis* is commonly implicated in Infective Endocarditis (IE), resulting in remarkable morbidity and mortality. We present an unusual case documenting the clinical course and outcome of an elderly female patient who developed quadruple valve endocarditis due to *Enterococcus faecalis* infection. She presented with altered mental status, resulting in hospitalization, and was found to have bacteremia complicated by endocarditis, epidural abscess, discitis, and splenic infarction. Urinalysis was consistent with bacterial infection two days before being admitted to the hospital. Unfortunately, despite aggressive therapeutic regimens, the patient died.

This is one of the few documented endocarditis cases involving all heart valves. It reviews the importance of maintaining a high index of clinical suspicion for assessing IE, with a low threshold for performing a transesophageal echocardiogram as a diagnostic tool.

## Introduction

*Enterococcus faecalis* is a gram-positive facultative anaerobe bacterium frequently found in the human gastrointestinal tract. It most often affects hospitalized individuals, causing bacteremia by direct translocation from the gut or via secondary bloodstream seeding from infections of intravenous lines and the urinary tract [1]. However, a small portion of patients have community-acquired infections. For example, as many as 10.2% of female community-acquired urinary tract infections (CAUTIs) can be caused by* E. faecalis* [2]. *Enterococcus faecalis* is the third most frequent (approximately 10% of cases) causative agent of infective endocarditis (IE) after *Staphylococcus* and *Streptococcus* [3], and commonly affects older and debilitated patients, causing significant in-hospital mortality.

Diagnosis of *Enterococcus faecalis *IE can be challenging, as patients often have a subacute course of disease with nonspecific symptoms and may be afebrile. In addition, resistance rates to commonly used antibiotics - such as ampicillin and vancomycin - have increased over the past few decades, posing significant obstacles to cure, and relapse or recurrence after treatment is not uncommon [4]. Overall, *E. faecalis *IE most commonly affects the aortic valve (56%), followed by the mitral valve (24%) [5], and the disease of all four native heart valves is exceedingly rare. 

## Case presentation

A 66-year-old woman presented to the emergency department (ED) with fatigue, shortness of breath, and moderate cramping lower abdominal pain. She was accompanied by a family member who had noticed decreased appetite, increasing somnolence, and urinary incontinence. Her previous medical history was significant for type 2 diabetes, liver cirrhosis due to non-alcoholic fatty liver disease (NAFLD), chronic kidney disease (CKD) stage IIIB, chronic obstructive pulmonary disease (COPD), and tobacco use disorder with a 30 pack-year history. This patient presented to the same ED two days before with similar concerns. On her first ED visit, the physical exam and laboratory tests were grossly unremarkable, although auscultation of the lungs was significant for mild expiratory wheezing. Laboratory investigations revealed elevated white blood cells (WBC). The urinalysis (UA) showed positive leukocyte esterase, elevated WBCs to 180 HPF, red blood cells (RBCs) of 26, negative nitrites, and one hyaline cast.

She was empirically treated for COPD exacerbation with one dose of inhaled albuterol and ipratropium (0.5 mg plus 3 mg per dose) plus intravenous methylprednisolone 125 mg. Ceftriaxone 2 grams was used to manage urinary tract infection (UTI). She was discharged in stable and improved condition with a 5-day prescription for oral cephalexin 500 mg four times daily and prednisone 40 mg daily for five days. Unfortunately, the patient's urinary incontinence and somnolence did not improve despite UTI treatment. On her second ED visit, two days afterward, she was afebrile, her blood pressure (BP) was 99/56 mmHg, her heart rate was 91 and regular, her pulse oximetry was 96% in room air, and her BMI was 46.7 kg/m2. She was alert and oriented but significantly somnolent, often falling asleep mid-sentence. The physical exam did not reveal any heart murmurs or acute skin changes.

The patient's workup was significant for creatinine and WBC elevation and low blood glucose of 41 mg/dL. The electrocardiogram (EKG) revealed normal sinus rhythm with no ST segment changes. Repeat UA showed no leukocyte esterase, white blood cells, or bacteria. Computerized tomography (CT) of the head without contrast was negative for any acute processes. CT of the abdomen and pelvis with contrast was concerning for air in the urinary bladder, suggestive of emphysematous cystitis, nonobstructive bilateral nephrolithiasis, retroperitoneal lymphadenopathy, and indeterminate spleen masses. Considering previous UA findings, cefepime 2g was started for empiric treatment of complicated UTI, and the patient was admitted to the hospital. Table 1 presents the results of the laboratory testing on the first and second ED visits.

**Table 1:** Laboratory testing on first and second ED visits A1C=glycosylated hemoglobin; BUN=blood urea nitrogen; BNP=B-type natriuretic peptide; AST=aspartate transaminase; ALT=alanine transaminase

Laboratory test	Initial ED visit, day 1	Second ED visit, day 3	Reference range
White blood cell	13.0 (11.1 neutrophils)	11.7 (10.4 neutrophils)	4.5-11 K/uL white blood cell count; 1.8-7.7 K/uL neutrophil absolute count
Hemoglobin	11.4	11.7	12-15 gm/dL
Hematocrit	34.1	33.3	36-46%
Platelets	203	282	150-400 K/uL
Glucose	257	41	69-111 mg/dL
A1C	8.6	-	4.0 – 6.0 %
BUN	50	76	8-21 mg/dL
Creatinine	1.7	1.9	0.4-1.2 mg/dL
Sodium	135	138	135-145 mmol/L
Potassium	5.2	6.4	3.5-5.1 mmol/L
BNP	432	-	0-100 pg/mL
Lactic acid	-	1.5	0.4-1.3 mmol/L
Ammonia	-	30	9-35 mcmol/L
AST	33	55	13-36 IUnits/L
ALT	32	47	6-40 IUnits/L
C-reactive protein	-	7.5	0.0 - 0.9 mg/dL
Urine culture	-	E. faecalis	No growth
Blood cultures	-	E. faecalis	No growth

**Figure 1:**
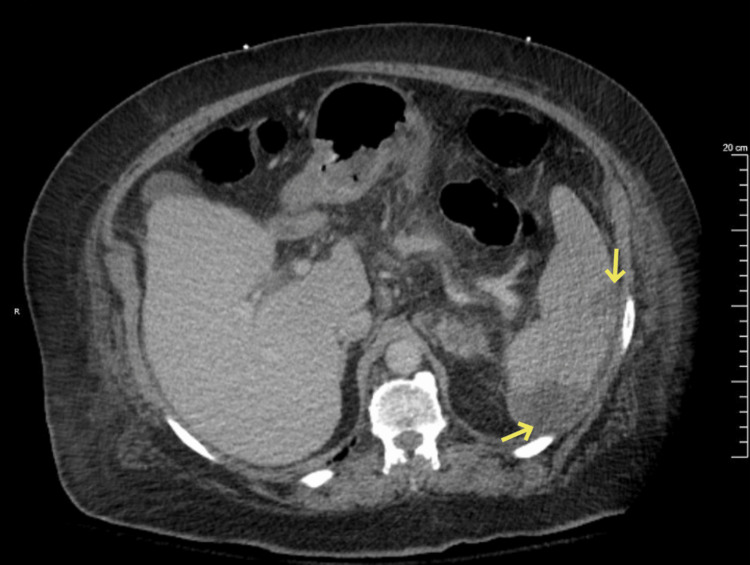
Computerized tomography of the abdomen and pelvis (with contrast) on transversal view, revealing lesions concerning for embolization of the spleen (yellow arrows).

On day two after admission to the Internal Medicine service, two peripherally obtained blood cultures from admission grew ampicillin- and vancomycin-susceptible *Enterococcus faecalis*. An infectious disease consult was requested, and the antibiotic regimen was transitioned to ampicillin 1 g every 6 hours. Urine culture from admission also grew the same agent with a similar susceptibility pattern. Regrettably, no urine culture had been obtained at her first ED visit. On day 2 of hospital admission, the patient had worsening lower abdominal pain, predominantly manifesting in the right lower quadrant without radiation. Repeat CT imaging of the abdomen and pelvis revealed several perfusion defects within the spleen suggestive of splenic infarcts (Figure 1). Concern for complicated bacteremia with embolization to the spleen prompted adding ceftriaxone 2 g a day to her regimen. There were no other imaging results that suggested any other causes of abdominal pain.

**Figure 2:**
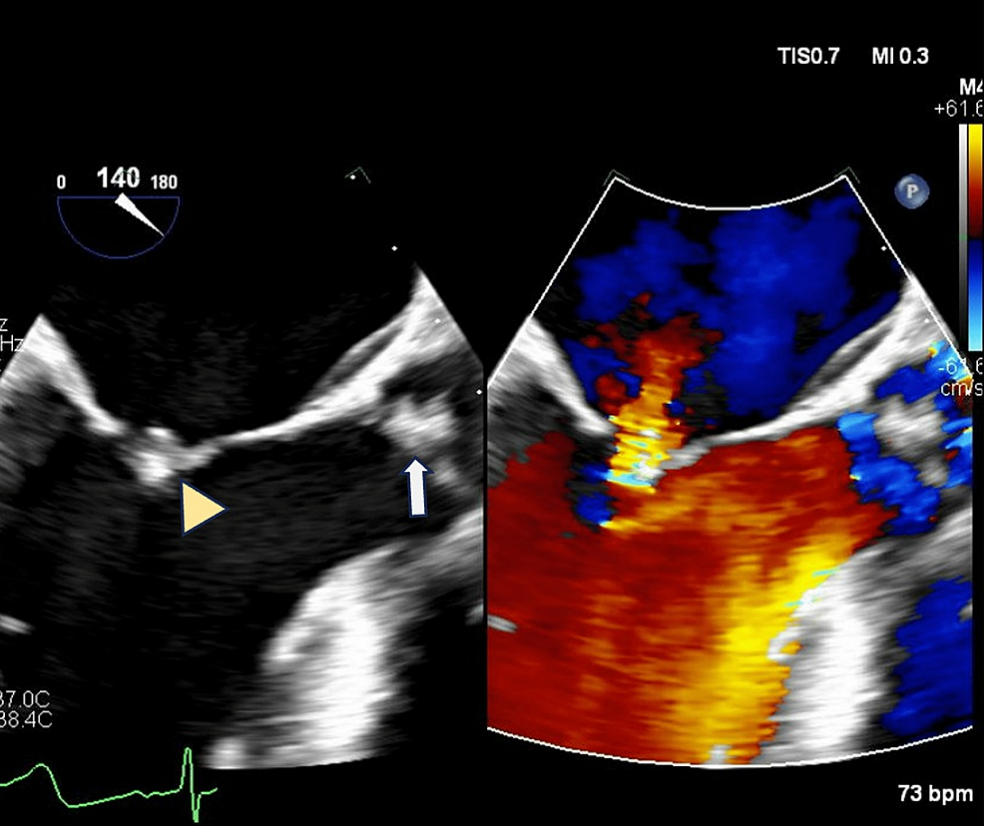
Transesophageal echocardiogram view of vegetation in mitral (arrowhead) and aortic (white arrow) valves. Doppler-colored blood flow through mitral valve is also shown in the figure.

Throughout the hospital stay, repeat blood cultures had no growth. The patient became progressively more oriented and conversant and seemed to have appropriate clinical and laboratory improvement. However, on day 6 of hospitalization, she reported severe thoracolumbar spine pain, stabbing in quality with a similar level as childbirth pain, and stated that it did not radiate. Magnetic resonance imaging (MRI) of the spine without contrast showed epidural fluid collection from T6 to L1 vertebras and another from L1 to sacrum, with discitis and osteomyelitis at L1-2 and L2-3. Neurosurgery was consulted and recommended conservative treatment. Following an unremarkable transthoracic echocardiogram, a transesophageal echocardiogram (TEE) was performed on day 8, revealing vegetation on the mitral, tricuspid, and aortic valves, with possible small vegetation on the pulmonic valve. The mitral vegetation was 1.2 x 0.9 centimeters (cm), and the aortic vegetation was 0.9 x 0.8 cm, with smaller lesions on the pulmonic and tricuspid valves (Figure 2). The cardiothoracic surgery team was consulted, but the patient was deemed a poor surgical candidate due to her concurrent bacteremia and COPD history.

On day 9 of admission, the patient had an unanticipated episode of aspiration while eating her dinner. She had no history of dysphagia. Despite initially feeling well with stable vital signs, the patient progressed to rapid decompensation with increased work of breathing, hypotension with BP of 57/40 mmHg, and peripheral pulse oximetry down to 75%. She required endotracheal intubation and was transferred to the Intensive Care Unit (ICU) in severe condition. She underwent urgent bronchoscopy with the removal of a mucous plug from the left mainstem bronchus. The patient developed worsening hemodynamic shock despite multiple vasopressors, continued antibiotic therapy, fluid resuscitation, and stress dose steroids. Bacterial cultures and gram-stain of bronchoalveolar fluid were negative. The patient became progressively more acidotic due to shock, with deterioration of renal function. The palliative care department discussed her condition and prognosis with her family, who chose comfort-focused care. The patient was extubated and passed away on day 10 of hospital admission. An autopsy was not performed.

## Discussion

*Enterococcus faecalis* bacteremia is associated with high mortality rates, and many complications are related to it, mainly multi-organ embolization and IE. Chirouze et al. [3] reveal that patient variables associated with increased 1-year mortality of IE include a diagnosis of heart failure, advanced age, and stroke. The same study also shows that IE due to *Enterococcus* species (which includes *E. faecium*) is often developed with healthcare intervention and in elderly patients with co-morbid conditions. Several risk factors predict an endocarditis diagnosis in *E. faecalis *bacteremia - which can occur in as much as 26% of bacteremic patients - including equal or more than three positive blood cultures, monomicrobial infection, community acquisition, prosthetic heart valve, an unknown portal of entry, or urinary tract infection [5].

As with other causes of IE in non-intravenous drug users, right-sided valves are rarely affected. The aortic valve is the most commonly involved in *E. faecalis *bacteremia, followed by mitral or combined aortic and mitral [5]. There is a remarkable lack of reported cases of quadruple valve endocarditis due to this bacterium. In our literature review, there have been only three reported cases in the last 20 years [6,7,8]. One 64-year-old man with a history of type II diabetes mellitus, chronic renal failure (not on hemodialysis), and hypertension was diagnosed with post-operative hospital-acquired *E. faecalis *bacteremia after a well-succeeded cervical laminectomy procedure [6]. The presentation for this individual was atypical, as neurological procedures are not a well-established risk factor for *E. faecalis *bacteremia [1, 6]. The patient was on gentamicin and vancomycin treatment but died despite medical treatment. Another report is of a 76-year-old man with chronic renal disease and factor XIII deficiency presenting with fever, fatigue, and acute heart failure, who had one of the first documented successful quadruple valve replacements for acute endocarditis in one operation [7]. In that scenario, simultaneous *E. faecalis *and *G. morbillorum *infections were culprits. Finally, a publication from 2013 showed complete recovery of quadruple valve endocarditis in a 16-year-old girl with a 6-week course of imipenem and linezolid [8]. This patient was diagnosed after presenting with a fever and weight loss for six months. This case was unique because there was no previous pathological medical history and no identifiable cause for immunosuppression.

It is crucial to address concerns of IE promptly with high-quality imaging. Dahl et al. [5] suggest that TEE should be considered for all patients with *E. faecalis *bacteremia as a part of the assessment, as in their cohort with 344 bacteremic patients, transthoracic echocardiography missed vegetation in 47% of the cases. Notably, TEE has higher sensitivity for detecting vegetation than transthoracic echocardiogram and can detect left-sided lesions as small as 2-5 mm with a sensitivity of 94% to 100% and 77% to 95% of specificity for native and prosthetic valves [9]. However, this is an invasive procedure with high associated costs, and it might not be appropriate for all patients with bacteremia. One cohort published in 2019 suggests that only patients with certain risk factors for IE in the setting of *E. faecalis *bacteremia should have a TEE and proposed the DENOVA score, which includes the presence of the following variables: duration of symptoms >7 days, embolization, > two positive blood cultures, the unknown origin of infection, previous valve disease and auscultation of murmur. A sensitivity of 100% and specificity of 83% were noted when three or more variables were present [10]. Despite this being a retrospective study with a limited sample size, other authors have found the DENOVA score an appropriate screening tool for IE - even when caused by other bacteria [11,12].

Our patient did have several days of symptoms, in addition to findings consistent with embolization and two positive blood cultures. Her TTE was negative for endocarditis, but TEE performed days later was diagnostic. Repeat TEE imaging after negative transthoracic or transesophageal imaging is strongly recommended if there is high suspicion for IE [4,10]. Of note, the well-established modified Duke criteria for IE diagnosis classifies TEE findings as a major criterion for diagnosis, recommending it early for patients with prosthetic valves. However, TTE should be used as a first test in other patients [13].

There are no established differences regarding treatment strategies when comparing quadruple with one or two valve endocarditis due to *E. faecalis*. Indications for valve replacement surgery in IE of native valves - regardless of causative agent - include failure to clear blood cultures after 5-7 days of antibiotic therapy, large mobile vegetations (>10 mm) with clinical embolic phenomena, and local complications such as para-valvular abscess, heart blocks, or destructive penetrative lesions [14]. In this case report, the patient had a significant size of mitral valve vegetation of >1 cm, and findings were consistent with embolization of the spleen. Unfortunately, in-hospital mortality for valve replacement surgery can be as high as 20%, and in *E. faecalis *infection, it is not associated with improved survival [3,15]. Therefore, this was not indicated for our patient as co-morbidities such as COPD and cirrhosis posed a very high risk. Instead, she received a combination therapy of ampicillin and ceftriaxone. Despite ampicillin being a first-line drug for non-resistant bacteria, synergistic agents such as cephalosporins and aminoglycosides are commonly added for increased effect in bacteremic and critically ill patients [16].

Although an autopsy was not performed, TEE imaging was consistent with the disease of all four valves. Remarkably, false-positive findings can occur in some scenarios, such as when strands are observed, which is especially important for patients with prosthetic valves [17]. In addition, several other factors could mimic endocarditis, including degenerative valvular tissue, thrombus, flail chords, or artifacts from calcium or prosthetic material [9]. However, clinical signs and symptoms of bacteremia with associated embolization and positive blood cultures should assist physicians in establishing a diagnosis.

## Conclusions

*Enterococcus faecalis *bacteremia can be devastating, often resulting in IE. Clinical signs and symptoms are vague and general work-up can be non-specific. Therefore, clinicians should maintain a high suspicion of endocarditis in such patients. TEE is an indispensable tool for analyzing and diagnosing native valve endocarditis. Scores such as DENOVA help facilitate medical decisions on obtaining this imaging.

Endocarditis rarely comprises all four valves, and despite being an unusual diagnostic entity, antimicrobial therapy and surgical treatment do not currently differ from patients with less affected valves. This report will hopefully contribute to knowledge on the topic given the scarce literature.
